# Prevalence and characteristics of rapid eye movement sleep behaviour disorder in adults with migraine: a cross‐sectional screening study

**DOI:** 10.1111/ene.16403

**Published:** 2024-07-05

**Authors:** Kristin Sophie Lange, Jasper Mecklenburg, Lucas Hendrik Overeem, Mira Pauline Fitzek, Anke Siebert, Maureen Steinicke, Paul Triller, Lars Neeb, Jens Peter Dreier, Daniel Kondziella, Uwe Reuter, Bianca Raffaelli

**Affiliations:** ^1^ Department of Neurology Charité—Universitätsmedizin Berlin, Corporate Member of Freie Universität Berlin, Humboldt‐Universität zu Berlin, and Berlin Institute of Health Berlin Germany; ^2^ Clinician Scientist Program Berlin Institute of Health at Charité (BIH) Berlin Germany; ^3^ Helios Global Health Berlin Germany; ^4^ Center for Stroke Research Charité—Universitätsmedizin Berlin, Corporate Member of Freie Universität Berlin, Humboldt‐Universität zu Berlin, and Berlin Institute of Health Berlin Germany; ^5^ Department of Experimental Neurology Charité—Universitätsmedizin Berlin, Corporate Member of Freie Universität Berlin, Humboldt‐Universität zu Berlin, and Berlin Institute of Health Berlin Germany; ^6^ Bernstein Center for Computational Neuroscience Berlin Berlin Germany; ^7^ Einstein Center for Neurosciences Berlin Berlin Germany; ^8^ Department of Clinical Medicine University of Copenhagen Copenhagen Denmark; ^9^ Department of Neurology Rigshospitalet, Copenhagen University Hospital Copenhagen Denmark; ^10^ Universitätsmedizin Greifswald Greifswald Germany

**Keywords:** headache, migraine, sleep, REM sleep behaviour disorder

## Abstract

**Background and purpose:**

Migraine and sleep disorders share a bidirectional relationship, but little is known about the specific association between migraine and rapid eye movement (REM) sleep behaviour disorder (RBD). The aim was to assess the prevalence of RBD and associated clinical characteristics in adults with migraine.

**Methods:**

This analysis is part of a cross‐sectional survey study conducted at the Headache Centre of the Charité—Universitätsmedizin Berlin between August 2020 and March 2023. At the end of their regular medical consultation, patients with migraine filled out (1) the validated RBD Screening Questionnaire (RBDSQ), (2) a questionnaire on REM sleep intrusions and (3) the Depression, Anxiety and Stress Scale 21. The primary endpoint was the percentage of patients with a positive RBD screening. A multivariate analysis was performed to identify characteristics independently associated with features of RBD.

**Results:**

A total of 751 patients (44.1 ± 13.2 years; 87.4% female) with complete RBDSQ were included in this analysis, of which 443 (58.9%) screened positive for RBD. In multivariate analysis, a positive screening for RBD was associated with younger age (odds ratio [OR] 0.9, 95% confidence interval [CI] 0.8–0.9 per 10‐year increase; *p* = 0.005) and with features suggestive of REM sleep intrusions (OR 4.3, 95% CI 1.8–10.4; *p* = 0.001). Migraine aura remained in the model without reaching statistical significance (OR 1.3, 95% CI 0.9–1.8; *p* = 0.079).

**Discussion:**

Symptoms of RBD are frequent in adults with migraine. Further studies including polysomnography are required to confirm this association, and to explore potential common pathophysiological mechanisms.

## INTRODUCTION

Migraine is one of the most frequent primary headache disorders, impacting more than 1 billion individuals worldwide [1]. Patients with migraine report poor sleep quality significantly more often compared to healthy individuals [2], and sleep disorders are frequent comorbidities of migraine, affecting approximately 48% of patients [3]. The relationship between migraine and sleep is bidirectional with an irregular sleep–wake pattern and low sleep quality as a potential trigger of migraine attacks and vice versa migraine attacks as a potential reason for sleep disturbances [2]. In addition, one genome‐wide association study provided evidence for a shared genetic susceptibility between migraine and sleep disorders [4]. The investigation of sleep disorders in migraine is important, since migraine itself is a highly disabling disease and a cardiovascular risk factor [5, 6], and comorbid sleep disorders might lead to additional worsening of quality of life and of cardiovascular health [7, 8].

Until now, most studies on migraine and sleep disorders have focused on insomnia, central disorders of hypersomnolence, sleep‐related breathing disorders and sleep‐related movement disorders, whilst only one study has assessed prevalence and features of rapid eye movement (REM) sleep behaviour disorder (RBD) [2, 9]. RBD belongs to the spectrum of parasomnias and is characterized by a loss of the physiological REM sleep skeletal muscle atonia, leading to dream‐enacting behaviour [10]. The diagnosis is made based on clinical and polysomnographic criteria according to the International Classification of Sleep Disorders, 3rd edition (ICSD‐3) [11]. Several studies point to a link between migraine and REM sleep, with alterations of REM sleep patterns [12, 13, 14], and indirect evidence for a higher prevalence of REM sleep intrusions in patients with migraine compared to the general population [15, 16]. Pathophysiologically, the thalamus, hypothalamus and the brainstem play a role both in the generation of migraine attacks and in REM sleep regulation [17, 18].

Psychiatric disorders are another frequent comorbidity of both migraine and sleep disorders [19, 20]. In more than 50% of patients with migraine, symptoms of anxiety and depression occur at least once in their lifetime [21, 22]. In patients with a psychiatric diagnosis, the risk of RBD is 10‐fold higher compared to the general population [23].

Given the pathophysiological and epidemiological indications for a link between migraine and REM sleep, on the one hand, and the evidence for a high burden of sleep disorders in patients with migraine, on the other hand, the aim of our study was to assess the prevalence of RBD symptoms in a large cohort of patients with migraine. Moreover, the aim was to assess an association of signs of RBD with demographic characteristics, migraine clinical features, REM sleep intrusions and symptoms of depression, anxiety and stress.

## METHODS AND MATERIALS

### Study design and participants

This work is part of a cross‐sectional survey study conducted at the Headache Centre, Charité—Universitätsmedizin Berlin, Germany. The design has been described in detail in the primary publication [15]. Briefly, from August 2020 to March 2023, adult patients with a confirmed diagnosis of migraine according to criteria of the International Classification of Headache Disorders, 3rd edition (ICHD‐3) [24] were invited to participate in this survey after their regular visit at our outpatient clinic. Exclusion criteria were other headache disorders except tension‐type headache, polypharmacy defined as more than five medications on a regular basis, insufficient German language proficiency, and severe psychiatric disorders impairing the completion of a standardized questionnaire. This study is reported in accordance with the Strengthening the Reporting of Observational Studies in Epidemiology (STROBE) statement for cohort studies.

### Study instruments

The survey questionnaire was filled out on an iPad on site. The questionnaire subsections relevant to this analysis include (i) demographic and headache characteristics, (ii) REM Sleep Behaviour Disorder Screening Questionnaire (RBDSQ) [25], (iii) REM sleep intrusions and (iv) Depression, Anxiety and Stress Scale 21 (DASS‐21). The REDCap application (Research Electronic Data Capture, Vanderbilt University, Nashville, TS, USA) was employed to collect all data in an electronic database.

#### Demographic and headache characteristics

Study staff entered information on age, biological sex, migraine diagnosis (episodic or chronic, with aura or without aura), monthly headache days (MHD), monthly migraine days (MMD) during the past 28 days, and current medication for migraine preventive treatment and for comorbidities.

The iPad was then given to the study participants who completed the remaining questionnaire sections with assistance of medical staff if needed. The headache survey for patients comprised questions related to disease duration, family history for migraine and the type of migraine aura in the case of migraine with aura.

#### Rapid Eye Movement Sleep Behaviour Disorder Screening Questionnaire

The RBDSQ is a self‐administered questionnaire to assess clinical features indicative of RBD [25]. It consists of 10 items, which have to be answered by either ‘yes’ or ‘no’ with a maximum total score of 13 points. A total score ≥5 points indicates a positive screening test for RBD. For the purpose of this study, item 10 ‘I have a disease of the nervous system’ was omitted from the questionnaire, but all patients received 1 point for this item for the diagnosis of migraine. The German RBDSQ version has been validated by polysomnography with a sensitivity of 0.96 and a specificity of 0.56 for RBD [25]. The RBDSQ was used to determine the prevalence of a positive RBD screening. A positive screening result does not confirm the diagnosis of RBD, which needs confirmation of REM sleep atonia by video polysomnography [11].

#### Rapid eye movement sleep intrusions

The REM sleep intrusion questionnaire by Nelson et al. [26] is a self‐administered questionnaire to assess the four features of (1) visual and (2) acoustic hypnagogic or hypnopompic hallucinations, (3) sleep paralysis and (4) sudden muscle weakness suggestive for cataplexy. The questionnaire was translated into German applying a standard forward–backward translation procedure as stated previously [15]. Screening for REM sleep intrusions was defined as positive if patients fulfilled three or more of the four criteria (a) visual and (b) acoustic hypnagogic or hypnopompic hallucinations, (c) sleep paralysis and (d) sudden muscle weakness suggestive for cataplexy.

#### Depression, Anxiety and Stress Scale 21

The DASS‐21 is a self‐administered questionnaire to assess the presence and severity of symptoms of depression, anxiety and stress [27, 40, 41]. It consists of three seven‐item scales with a total of 21 items, which are rated on a 4‐point Likert scale from 0 ‘Does not apply to me at all’ to 3 ‘Applies to me very much or most of the time’. According to predefined cut‐off values, depression, anxiety and stress were rated as mild, moderate, severe or extremely severe, respectively [28]. The DASS‐21 German version has been shown to provide good reliability and validity [29].

### Endpoints and objectives

The primary endpoint for this study was the prevalence of a positive RBD screening in patients with migraine, defined as the percentage proportion of patients with positive scores for RBD in the RBDSQ. Secondary objectives were to compare patients with versus patients without a positive RBD screening with regard to (1) demographic and headache characteristics, (2) features indicative of REM sleep intrusions and (3) symptoms of depression, anxiety and stress. Exploratory analyses included the correlation between the RBDSQ scores and age, MHD and MMD, and the DASS‐21 scores. To improve readability, patients who screened positive for RBD were labelled *patients with RBDSQ+* and patients who screened negative for RBD *patients with RBDSQ*− throughout the results section.

### Statistical analyses

Statistical analyses were performed with SPSS 27.0 (IBM SPSS Statistics©, Armonk, NY, USA). Missing values are indicated for each analysis. Categorical variables are reported as absolute numbers (*n*) and percentages (%), numerical variables as mean value ± standard deviation. Due to the large sample size, tests were not performed for normal distribution. Differences in continuous variables were determined using the Student *t* test. Categorical variables were compared between two groups by Pearson's chi‐squared test or the Fisher exact test. Correlations were assessed using Pearson correlation analyses. To determine independent associations with a positive RBD screening, a stepwise backward logistic regression was performed for estimation of multivariable odds ratios (ORs) and their 95% confidence interval (CI), with adjustment for variables with significant differences between the groups in univariate analysis. Criteria for inclusion into the model and remaining in the model were *p* < 0.05 and *p* < 0.10, respectively. A two‐tailed *p* value ≤0.05 was considered as statistically significant. Since this is a hypothesis‐generating study, no adjustments for multiple testing were made.

### Ethical standards

The study was approved by the ethics committee of the Charité—Universitätsmedizin Berlin (EA1/149/20) and registered in the German Clinical Trials Registry (Deutsches Register Klinischer Studien) with the ID DRKS00025845. All participants provided written informed consent prior to their participation in the survey.

## RESULTS

Of 808 patients with migraine who completed the survey, 751 patients with complete RBDSQ were included in this analysis (Table 1). Mean age was 44.1 (± 13.2) years. There was a female preponderance (656 women, 87.4%). The majority of patients had a diagnosis of episodic migraine (*n* = 561, 74.7%), with a mean number of MHD and MMD of 11.5 (± 7.2) and 8.8 (± 6.0) respectively in the overall cohort. In all, 325 patients (43.3%) had migraine with aura, with visual aura symptoms in 288 (38.3%), sensory symptoms in 136 (18.1%) and speech impairment in 137 (18.2%) patients. Almost half of the patients currently received a migraine preventive medication (46.1%), with the most frequent prophylactic treatment being monoclonal antibodies against calcitonin gene‐related peptide or its receptor. Of 71 patients (8.8%) on serotonergic antidepressants and 83 patients (10.3%) on beta‐blockers, five patients (0.7%) and 58 (7.7%) respectively were taking them for the indication migraine.

**TABLE 1:** Demographic and headache characteristics of the overall cohort; comparison of patients with positive and negative RBDSQ.

Variable	All (*n* = 751)	RBDSQ+ (*n* = 443)	RBDSQ− (*n* = 308)	*p* value
Sex (female)	656 (87.4)	390 (88.0)	266 (86.4)	0.498
Age (years)	44.1 (13.2)	42.9 (13.0)	44.9 (13.3)	**0.002**
Migraine characteristics				
Chronic migraine	190 (25.3)	108 (24.4)	82 (26.6)	0.487
Family history	524 (69.8)^a^	307 (69.3)	217 (70.5)	0.676
Years since first migraine manifestation	23.7 (13.7)^b^	23.1 (13.4)	24.5 (14.1)	0.177
Years since migraine diagnosis	15.2 (12.8)^c^	14.9 (12.7)	15.6 (13.0)	0.478
MHD	11.5 (7.2)^d^	11.7 (7.1)	11.2 (7.5)	0.333
MMD	8.8 (6.0)^e^	8.6 (5.7)	8.9 (6.5)	0.479
Migraine with aura	325 (43.3)	205 (46.3)	120 (39.0)	**0.047**
Visual symptoms	288 (38.3)	184 (41.5)	104 (33.8)	**0.031**
Sensory symptoms	136 (18.1)	88 (19.9)	48 (15.6)	0.134
Speech impairment	137 (18.2)	92 (20.8)	45 (14.6)	**0.032**
Migraine preventive treatment				
Any	346 (46.1)	201 (45.4)	145 (47.1)	0.645
CGRP (‐receptor) monoclonal antibodies	144 (19.2)	83 (18.7)	61 (19.8)	0.714
Antidepressants	50 (6.7)	33 (7.4)	17 (5.5)	0.297
Tricyclic antidepressant	47 (5.8)	30 (6.8)	15 (4.9)	0.280
Serotonergic antidepressant	5 (0.7)	3 (0.7)	2 (0.6)	0.963
Beta‐blockers	58 (7.7)	30 (6.8)	28 (9.1)	0.242
Flunarizin	18 (2.4)	13 (2.9)	5 (1.6)	0.248
Antiseizure medication	37 (4.9)	22 (5.0)	15 (4.9)	0.952
OnabotulinumtoxinA	62 (8.3)	38 (8.6)	24 (7.8)	0.700
Other	28 (3.7)	17 (3.8)	11 (3.6)	0.850
Medication with potential influence on REM sleep				
Antidepressants for any indication	126 (15.6)	80 (18.1)	35 (11.4)	**0.012**
Serotonergic antidepressant	71 (8.8)	45 (10.2)	17 (5.5)	**0.023**
Beta‐blockers for any indication	83 (10.3)	40 (9.0)	35 (11.4)	0.294

*Note*: Values are given as mean (± standard deviation) for continuous data and as *n* (%) for categorical data. *p* value from Pearson *χ* ^2^ test for categorical variables, Student *t* test for continuous variables. Bold values denote statistical significance at the *p* < 0.05 level. Missing data for *n* (%) patients: ^a^1 (0.1), ^b^34 (4.5), ^c^43 (5.7), ^d^26 (3.5), ^e^32 (4.3).

Abbreviations: CGRP, calcitonin gene‐related peptide; MHD, monthly headache days; MMD, monthly migraine days; RBDSQ, REM Sleep Behaviour Disorder Screening Questionnaire; RBDSQ+, patients screening positive for RBD; RBDSQ−, patients screening negative for RBD; REM, rapid eye movement.

### Prevalence and features of RBD in the overall cohort

**FIGURE 1:**
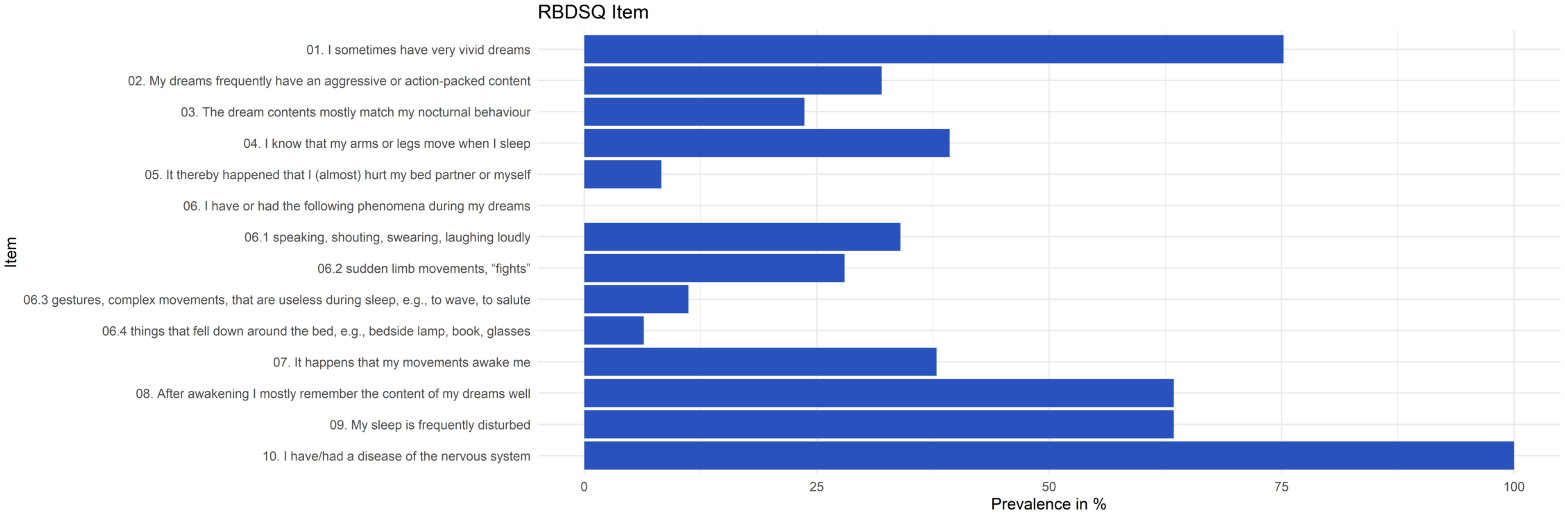
Frequency (%) of reported RBDSQ features in the overall cohort (*n* = 751). RBDSQ, REM Sleep Behaviour Disorder Screening Questionnaire.

The mean RBDSQ score in the overall cohort was 5.2 (± 2.4), and 443 (58.9%) were patients with RBDSQ+. The items most frequently reported were vivid dreams, remembering dream content very well, and frequently disturbed sleep (Figure 1).

### Comparison of patients with RBDSQ+ and RBDSQ−

#### Demographic characteristics and headache features

Table 1 displays the comparison of demographic characteristics and headache features between patients with RBDSQ+ and RBDSQ−. Patients with RBDSQ+ were significantly younger than patients with RBDSQ− (42.9 years ±13.0 vs. 44.9 years ±13.3, *p* = 0.002), and RBDSQ scores were negatively correlated with age (*r* = −0.11, *p* = 0.002). Whilst the percentage of patients with chronic migraine was similar between the groups, there was a positive correlation of the RBDSQ with the number of MHD (*r* = 0.083, *p* = 0.026) but not the number of MMD in the month prior to data collection.

Patients with RBDSQ+ had significantly more often a diagnosis of migraine with aura (46.3% vs. 39.0%, *p* = 0.047) compared to patients with RBDSQ−. With regard to subtypes of aura symptoms, patients with RBDSQ+ had more often visual symptoms (41.5% vs. 33.8%, *p* = 0.031) and speech impairment (20.8% vs. 14.6%, *p* = 0.032), whilst the rate of sensory symptoms was equal for both groups.

The proportion of patients with a migraine preventive treatment did not differ between patients with RBDSQ+ and patients with RBDSQ−, also when taking into account the type of prophylactic treatment.

### Rapid eye movement sleep intrusions

Of 744 patients with a complete questionnaire, 43 (5.7%) scored positive for REM sleep intrusions. Patients with RBDSQ+ reported significantly more often signs indicative of visual or acoustic hypnagogic or hypnopompic hallucinations, sleep paralysis and sudden muscle weakness (*p* < 0.001, respectively), and scored positive for REM sleep intrusions in 37 cases (8.4%) compared to six cases (2.0%) in the remaining sample (*p* < 0.001) (Table 2).

**TABLE 2:** Prevalence of REM sleep intrusions in participants with and without positive RBDSQ screening.

REM intrusion	All	RBDSQ+ (*n* = 438)	RBDSQ− (*n* = 306)	*p* value
Hypnagogic or hypnopompic hallucinations				
Visual hallucinations	74 (9.9)^a^	62 (14.0)	12 (3.9)	**<0.001**
Acoustic hallucinations	84 (11.2)^b^	73 (16.5)	11 (3.6)	**<0.001**
Sleep paralysis	177 (23.6)^b^	134 (30.4)	43 (14.0)	**<0.001**
Sudden muscle weakness	185 (24.6)^b^	131 (29.7)	54 (17.6)	**<0.001**
Screening positive (≥3 points)	43 (5.7)^c^	37 (8.4)	6 (2.0)	**<0.001**

*Note*: Values are given as *n* (%). *p* value from Pearson *χ* ^2^ test or Fisher exact test. Bold values denote statistical significance at the *p* < 0.05 level. Missing data for *n* (%) patients: ^a^2 (0.3), ^b^3 (0.4), ^c^7 (0.9).

Abbreviations: RBDSQ, REM Sleep Behaviour Disorder Screening Questionnaire; RBDSQ+, patients screening positive for RBD; RBDSQ−, patients screening negative for RBD; REM, rapid eye movement.

### Depression, anxiety and stress

The DASS‐21 questionnaire was completed by 749 of the 751 participants. The percentage of patients with symptoms of depression, anxiety and stress was significantly higher amongst patients with RBDSQ+ (49.1% vs. 30.9%, 52.7% vs. 27.0%, 50.7% vs. 21.2%, respectively; *p* < 0.001). When considering different grades of mild to extremely severe symptoms, patients with RBDSQ+ had higher percentages for all degrees of severity (Table 3). RBDSQ scores were positively correlated with all three dimensions of the DASS‐21, with the highest correlation between RBDSQ and anxiety (*r* = 0.358, *p* < 0.001), followed by stress (*r* = 0.356, *p* < 0.001) and depression (*r* = 0.247, *p* < 0.001).

**TABLE 3:** Symptoms of depression, anxiety and stress according to the DASS‐21; comparison of patients with positive and negative RBDSQ.

Variable	All (*n* = 749)	RBDSQ+ (*n* = 442)	RBDSQ− (*n* = 307)	*p* value
Depression				
Any	312 (41.7)	217 (49.1)	95 (30.9)	**<0.001**
Mild	94 (12.6)	59 (13.3)	35 (11.4)	
Moderate	123 (16.4)	90 (20.4)	33 (10.7)	
Severe	50 (6.7)	36 (8.1)	14 (4.6)	
Extremely severe	45 (6.0)	32 (7.2)	13 (4.2)	
Anxiety				
Any	316 (42.2)	233 (52.7)	83 (27.0)	**<0.001**
Mild	130 (17.4)	89 (20.1)	41 (13.4)	
Moderate	74 (9.9)	50 (11.3)	24 (7.8)	
Severe	55 (7.3)	46 (10.4)	9 (2.9)	
Extremely severe	57 (7.6)	48 (10.9)	9 (2.9)	
Stress				
Any	289 (38.6)	224 (50.7)	65 (21.2)	**<0.001**
Mild	100 (13.4)	68 (15.4)	32 (10.4)	
Moderate	105 (14.0)	85 (19.2)	20 (6.5)	
Severe	60 (8.0)	51 (11.5)	9 (2.9)	
Extremely severe	24 (3.2)	20 (4.5)	4 (1.3)	

*Note*: Values are given as *n* (%) for categorical data. *p* value from Pearson *χ* ^2^ test for categorical variables (comparison of any depression/anxiety/stress between two groups). Bold values denote statistical significance at the *p* < 0.05 level. DASS‐21 grading according to the user manual.

Abbreviations: DASS‐21, Depression, Anxiety and Stress Scale 21; RBDSQ, REM Sleep Behaviour Disorder Screening Questionnaire; RBDSQ+, patients screening positive for RBD; RBDSQ−, patients screening negative for RBD; REM, rapid eye movement.

### Multivariate analysis

After adjustment for variables associated with a positive RBD screening in univariate analysis, younger age and REM sleep intrusions were independently associated with RBD (Table 4). Migraine aura remained in the model, albeit without reaching statistical significance.

**TABLE 4:** Multivariate analysis on the association of clinical characteristics and a positive screening for RBD.

Outcome	Variable	OR (95% CI)	*p* value
RBDSQ+	REM sleep intrusions	4.3 (1.8–10.4)	0.001
	Age (per 10‐year increase)	0.9 (0.8–0.9)	0.005
	Migraine aura	1.3 (0.9–1.8)	0.079

*Note*: OR (95% CI) and *p* value from stepwise backward logistic regression including the variables age, REM sleep intrusions (positive screening), migraine aura, depression, anxiety or stress (according to the DASS‐21, respectively) and use of serotonergic depressants.

Abbreviations: CI, confidence interval; DASS‐21, Depression, Anxiety and Stress Scale 21; OR, odds ratio; RBDSQ, REM Sleep Behaviour Disorder Screening Questionnaire; RBDSQ+, positive screening for RBD; REM, rapid eye movement.

## DISCUSSION

In this cross‐sectional study including 751 patients with migraine, 58.9% of participants screened positive for RBD. Younger age and features suggestive of REM sleep intrusions were independently associated with a positive RBD screening.

Only one prior study has assessed the prevalence of RBD in patients with migraine. Suzuki et al. used the Japanese version of the RBDSQ to compare the prevalence of RBD features between 161 patients with episodic migraine and 140 control subjects [30]. The prevalence of a positive RBD screening was significantly higher in patients with migraine compared to controls (24.2% vs. 14.3%), but only half as high as in our study. This difference could have several reasons. First, headache frequency was lower in the Japanese study where patients with chronic migraine were not eligible for inclusion. This might have constituted a selection bias for patients with a lower burden of headache, which might be associated with a lower prevalence of comorbid sleep disorders [31]. Second, the percentage of patients with migraine with aura was much lower in the Japanese cohort, accounting for 24.2% opposed to 43.3% in our cohort, and a higher prevalence of a positive RBD screening was found in patients with migraine with aura. Third, mean age in the Japanese cohort was lower than in our cohort (33.1 ± 10.0 vs. 44.1 ± 13.2), and increased age is known to be one of the strongest risk factors for RBD [18]. As in our study, Suzuki et al. did not employ polysomnography to confirm a diagnosis of RBD. Several small studies employing polysomnography or nocturnal electroencephalography have reported altered REM sleep patterns in patients with migraine [12, 13, 14], but to our knowledge no studies using polysomnography to confirm or refute a diagnosis of RBD in patients with migraine have been conducted so far.

The prevalence of RBD confirmed by polysomnography is estimated at 0.5%–1.5% in the general population [18]. Prevalence estimates from studies using different screening questionnaires without confirmation by polysomnography indicate higher prevalences of 2.7%–13.4% [32, 33]. However, screening questionnaires are prone to detect other conditions including somnambulism or arousals from other causes [34]. In our study, a screening questionnaire was used for RBD without confirming the diagnosis by polysomnography. Whilst the true prevalence of RBD may have potentially been overestimated, the prevalence of a positive RBD screening remains approximately four times higher compared to the aforementioned studies conducted in the general population using identical or similar screening questionnaires. This indicates a higher rate of RBD in individuals with migraine.

Rapid eye movement sleep behaviour disorder occurs with various neurological diseases in which the inhibition of spinal motor neurons is disturbed by different mechanisms. The most common aetiologies of symptomatic RBD vary according to age, with α‐synucleinopathies such as Parkinson's disease being the most common cause in the elderly and narcolepsy or use of serotonergic antidepressant medication being the most common causes in younger patients [23, 35].

Concerning pathophysiological hypotheses for an association of migraine and RBD, the involvement of similar neuroanatomical structures and circuits might play a role. Structures involved in REM sleep regulation and inhibition of spinal cord motor neurons during REM sleep include the brainstem and hypothalamus, notably the sublaterodorsal nucleus and the ventral medulla, with activation from the ventrolateral periaqueductal grey and hypothalamic neurons [18]. Additionally, the activation of cortical structures including the motor cortex as well as the cortical and subcortical limbic system are supposed to contribute to the generation and, in RBD, the enactment of dream content [36, 37]. In migraine, the same or neighbouring structures are involved. Trigeminovascular neurons in the brainstem and in the hypothalamus are responsible for the transmission of nociceptive stimuli from the trigeminal ganglion to the cortex, resulting in the perception of migraine pain [38]. In migraine with aura, cortical spreading depolarization facilitated by cortical hyperexcitability leads to aura symptoms [39].

Neurodegenerative mechanisms involved in RBD secondary to α‐synucleinopathies are unlikely to be relevant for the pathophysiology of RBD in patients with migraine. First, in our cohort, younger age was independently associated with a positive RBD screening. Second, although there is some evidence for an elevation of serum total‐tau protein in patients with migraine compared to healthy controls, this is most likely attributable to inflammatory and not neurodegenerative mechanisms [40]. Third, studies on the association of migraine and other diseases have pointed to a higher prevalence of migraine in patients with basal ganglia disorders, but not a higher risk to develop α‐synucleinopathies in patients with migraine [41].

An independent association of REM sleep intrusions was found with a positive RBD screening. REM sleep intrusions are typical symptoms of narcolepsy, a central disorder of hypersomnolence characterized by a disruption of sleep–wake regulation [42]. Narcolepsy is known to be associated on the one hand with an increased prevalence of RBD of up to 60% [43], and on the other hand with a two‐ to four‐ increased prevalence of migraine [16]. The association seems to be more pronounced for migraine with aura [44]. In our cohort, migraine aura was associated with a positive RBD screening without reaching statistical significance. Possibly, an increased cortical excitability and susceptibility to spreading depolarization might contribute to an association of aura and RBD [39]. One study examined the concomitance of narcolepsy, migraine and a positive RBD screening and found an increased prevalence of a positive RBD screening in patients with narcolepsy and migraine compared to those without headache [9]. Our results might support a tridirectional relationship between migraine, narcolepsy and RBD, although it must be emphasized that screening was only carried out for RBD and REM sleep intrusions without confirming the diagnoses of RBD and narcolepsy according to the ICSD‐3 [11].

A significantly higher prevalence of a positive RBD screening was found in patients with positive DASS‐21 scores for depression, anxiety or stress compared to patients without psychiatric comorbidities, and a higher proportion of patients taking serotonergic antidepressants in patients with a positive RBD screening. An association of depression and idiopathic RBD has also been found in a large multicentre case–control study [45], and serotonergic antidepressants might induce RBD [23, 35]. However, in multivariate analysis, stress, anxiety or depression and serotonergic antidepressants were not independently associated with RBD.

More rarely, beta‐blockers have been reported to induce RBD [46]. In our cohort, proportions of patients receiving beta‐blockers did not differ between patients with and without a positive RBD screening.

To our knowledge, this is the largest study on RBD in patients with migraine so far, with a sample size allowing for subgroup analyses for different migraine forms and psychiatric comorbidities. The diagnosis of migraine and its classification into episodic or chronic and with or without aura was made by neurologists specialized in headache, ensuring a high degree of diagnostic accuracy. Further, patients completed the self‐administered part of the questionnaires on site with available assistance of medical staff which is supposed to reduce bias compared to online‐based surveys [47, 48].

The main limitation is that the diagnosis of RBD was not confirmed by polysomnography, and thus the prevalence of RBD might be overestimated. The RBDSQ is a validated screening questionnaire with high sensitivity but moderate specificity, thus possibly yielding a substantial false positive rate. In‐laboratory video polysomnography is necessary to make the diagnosis of RBD by proof of REM sleep without atonia [11]. However, assuming a specificity of 0.56 for the RBDSQ, if only 56% of those patients in our cohort who screened positive actually had RBD, this would correspond to 248 patients (33%) which is still approximately 30× higher than the estimated prevalence in the general population [18]. Whilst only features of RBD and REM sleep intrusions were assessed, future studies should take into consideration quantity and quality of sleep, and assess for sleep disorders which might mimic or overlap with RBD, including obstructive sleep apnoea, non‐rapid eye movement parasomnias, periodic limb movement disorder and nocturnal seizures [49]. Further, all patients received 1 point on the RBDSQ for the presence of a neurological disease, but no differentiation was made between patients in whom migraine was the only neurological disease and patients with additional neurological conditions. Finally, our cohort consists of a highly selected population from a tertiary headache centre which might not be representative for the entirety of patients with migraine. Of note, the proportion of patients with aura was higher than in previous studies, but similar to results from another tertiary headache centre [50] and one cross‐sectional survey study [51]. Migraine with aura might be underdiagnosed, or patients might confuse other unspecific visual phenomena with visual aura [51].

The hypotheses generated from this study support the purpose and feasibility of a prospective polysomnography study to examine REM sleep in patients with migraine compared to healthy controls.

## CONCLUSIONS

In conclusion, a positive screening for RBD is frequent in patients with migraine, and is independently associated with younger age and features suggestive of REM sleep intrusions. Common pathophysiological mechanisms of migraine and REM sleep disturbance might involve functional or structural alterations in the thalamus, hypothalamus and brainstem. Based on the results of this screening study, our plan is to conduct a prospective polysomnographic study to confirm an association of migraine and RBD, and to explore potential common pathophysiological mechanisms.

## AUTHOR CONTRIBUTIONS

**Kristin Sophie Lange:** Conceptualization; investigation; writing – original draft; visualization; formal analysis. **Jasper Mecklenburg:** Conceptualization; writing – review and editing. **Lucas Hendrik Overeem:** Investigation; formal analysis; writing – review and editing; visualization. **Mira Pauline Fitzek:** Investigation; writing – review and editing. **Anke Siebert:** Investigation; writing – review and editing. **Maureen Steinicke:** Investigation; writing – review and editing. **Paul Triller:** Investigation; writing – review and editing. **Lars Neeb:** Supervision; writing – review and editing. **Jens Peter Dreier:** Conceptualization; writing – review and editing. **Daniel Kondziella:** Conceptualization; supervision; writing – review and editing. **Uwe Reuter:** Conceptualization; supervision; writing – review and editing. **Bianca Raffaelli:** Writing – original draft; project administration; conceptualization; investigation.

## CONFLICT OF INTEREST STATEMENT

KSL reports personal fees from Teva, Acticor Biotech; JM reports personal fees from Novartis; LHO has nothing to disclose; MF reports personal fees from Teva, Novartis; AS reports personal fees from TEVA, Novartis; MS reports personal fees from Abbvie; PT reports personal fees from AbbVie; LN reports personal fees from Abbvie, Lilly, Lundbeck, Newsenselab, Novartis, Perfood, TEVA and research funding from DFG, DLR, Lilly and Teva; JPD has nothing to disclose; DK reports personal fees from Wiley, and research funding from amongst others the Lundbeck Foundation, Novo Nordisk Foundation; UR reports personal fees from Amgen, Allergan, Abbvie, Lilly, Lundbeck, Novartis, Pfizer, Medscape, StreaMedUp, Springer, Teva and research funding from Novartis; BR reports research grants from Novartis and personal fees from Abbvie/Allergan, Eli Lilly, Lundbeck, Novartis, Teva.

## Data Availability

Anonymized data may be shared at the request of any qualified investigator for purposes of replicating procedures and results.
